# High throughput, high resolution selection of polymorphic microsatellite loci for multiplex analysis

**DOI:** 10.1186/1746-4811-1-3

**Published:** 2005-08-18

**Authors:** Nicholas C Cryer, David R Butler, Mike J Wilkinson

**Affiliations:** 1 School of Biological Sciences, University of Reading, Reading, Berkshire, RG6 6AS, UK; 2 Cocoa Research Unit, The University of West Indies, St. Augustine, Trinidad and Tobago

**Keywords:** Multiplex, Microsatellite, High Throughput, Fluorescent, Dinucleotide, High-Resolution, Allelic Ladder

## Abstract

**Background:**

Large-scale genetic profiling, mapping and genetic association studies require access to a series of well-characterised and polymorphic microsatellite markers with distinct and broad allele ranges. Selection of complementary microsatellite markers with non-overlapping allele ranges has historically proved to be a bottleneck in the development of multiplex microsatellite assays. The characterisation process for each microsatellite locus can be laborious and costly given the need for numerous, locus-specific fluorescent primers.

**Results:**

Here, we describe a simple and inexpensive approach to select useful microsatellite markers. The system is based on the pooling of multiple unlabelled PCR amplicons and their subsequent ligation into a standard cloning vector. A second round of amplification utilising generic labelled primers targeting the vector and unlabelled locus-specific primers targeting the microsatellite flanking region yield allelic profiles that are representative of all individuals contained within the pool. Suitability of various DNA pool sizes was then tested for this purpose. DNA template pools containing between 8 and 96 individuals were assessed for the determination of allele ranges of individual microsatellite markers across a broad population. This helped resolve the balance between using pools that are large enough to allow the detection of many alleles against the risk of including too many individuals in a pool such that rare alleles are over-diluted and so do not appear in the pooled microsatellite profile. Pools of DNA from 12 individuals allowed the reliable detection of all alleles present in the pool.

**Conclusion:**

The use of generic vector-specific fluorescent primers and unlabelled locus-specific primers provides a high resolution, rapid and inexpensive approach for the selection of highly polymorphic microsatellite loci that possess non-overlapping allele ranges for use in large-scale multiplex assays.

## Background

Microsatellite analysis using fluorescently labelled primers and capillary fractionation is the pre-eminent method for the genetic analysis of eukaryotic organisms. The approach is routinely used for many applications including forensic analysis [1], linkage mapping and association genetics [2], population genetics [3,4] and genetic analysis of diversity [5]. The need to screen microsatellite loci for polymorphism between genotypes within the target organism, and for their suitability in multiplex analysis, is an inevitable part of such efforts. The high cost of fluorescently labelled primers has meant that selection of microsatellite markers has typically relied on initial, low-resolution screens of unlabelled primers prior to high-resolution marker selection using labelled primers. However, the preliminary screen is inevitably crude and inefficient, making it either prone to error or reliant upon the more expensive high-resolution selection. There is therefore a need for a high throughput and high-resolution single-step method of selecting appropriate microsatellite markers for genetic studies [6].

For multiplex analysis, greatest efficiency is achieved when utilising many polymorphic loci possessing closely spaced, non-overlapping allelic ranges. Unexpected allelic range overlap between multiplexed microsatellite loci yields ambiguous alleles that may be misassigned to an inappropriate locus, compromising the integrity of the data set. One inevitable problem lies in the possibility that the screen does not encompass all alleles present in the population under study. Confidence in the definition of allelic ranges is invariably a function of the number and diversity of genotypes screened. There is therefore a balance between the desire to examine many individuals and the cost of doing so using fluorescently labelled primers. Thus, screening invariably becomes expensive as the number of genotypes tested grows, and as the number of discarded markers increases. Common approaches to selecting microsatellite markers for multiplex use include assembling panels from previously fluorescently characterised individual markers [7,8], and pre-screening markers on polyacrylamide gels utilising radioactivite labelling of PCR products [9]. Several authors have proposed low cost alternatives for preliminary screens using direct DNA staining following polyacrylamide gel electrophoresis [10-13]. Such strategies have merit, but are labour-intensive, cannot assign actual size ranges and generally lack the resolution required to accurately predict polymorphism in dinucleotide markers [10-13]. One methodology that is able to generate high resolution allelic ladders in a similar fashion to the method reported here is that of Oetting [14]. This method employs the use of locus specific primers tailed with generic sequence allowing a second round of labelled PCR and subsequent capillary fractionation. This method however suffers from a number of potential disadvantages relative to our method. The use of long oligonucleotides for PCR of genomic templates at below optimal annealing temperature allows for an increased frequency in the production non specific amplification products. The PCR amplification conditions required for locus specific amplification using tailed oligonucleotides are often different to those conditions optimal for amplification with equal length 20 mer oligonucleotides. The method of Oetting is not suited to the genotypic analysis of dinucleotide repeat markers due the possibility of extensive stutter profiles generated by the second round of PCR complicating the allelic profiles and so is only considered of merit for marker selection.

Here, we propose a simple but novel approach in which microsatellite amplicons generated from pooled genomic DNA templates are ligated into a standard cloning vector, re-amplified using a labelled universal primer targeting the plasmid insert flanking region, and an unlabelled locus-specific primer. The resultant profiles represents allelic ladders derived from the component alleles contained by the pooled DNA. This procedure thereby offers a single assay, high resolution and inexpensive means of screening microsatellite loci for polymorphism and allelic size range. The profile also offers a qualitative indication of the locus with regard to stutter, a problem often associated with the use of dinucleotide repeat markers for genetic analysis, but also of interest to laboratories utilising tri- and tetranucletide repeat markers.

## Results

**Figure 1:**
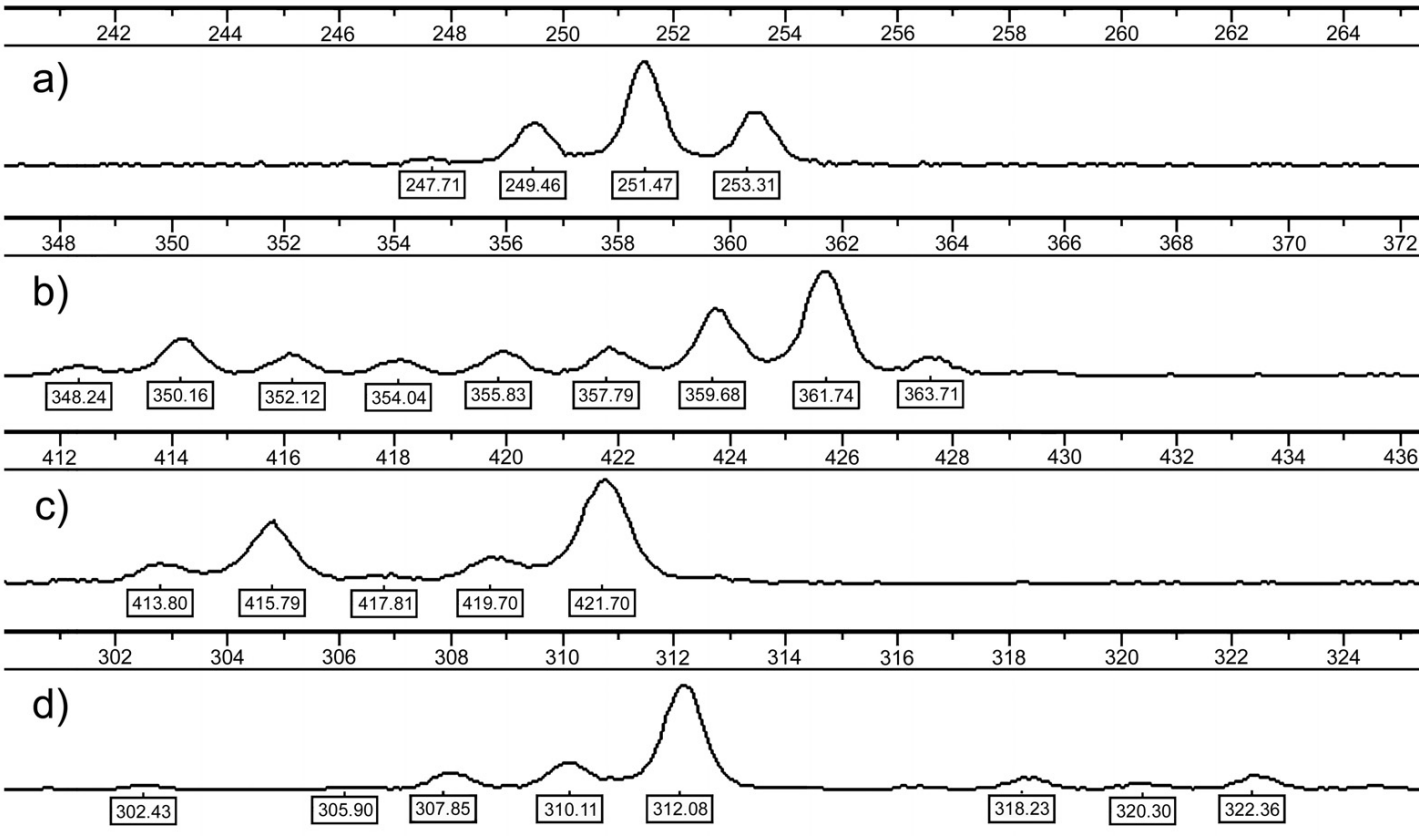
**Representative capillary electrophoresis traces for allelic screening of dinucleotide microsatellite loci against pooled DNA samples**. Microsatellite loci are A) mTcCIR080; B) mTcCIR131; C) mTcCIR155; D) mTcCIR190.

When employing a pooling strategy, there is a balance between sampling extensively to encompass the full range of variation, and dilution of individuals within the pool such that rare alleles are not detected. DNA pools were created by combining equal amounts of individual DNA samples before dilution with nano-pure water to 5 ng·μL^-1^. To select the most appropriate pool size, while allowing the detection of rare alleles, DNA pools of 8, 12, 16, 24, 32, and 48 individuals were compared. PCR amplification of pooled DNA utilising unlabelled primer pairs specific to single microsatellite loci [15] were performed incorporating 5 ng template DNA with AccuPrime *Taq *DNA Polymerase in supermix I, using half recommended volumes (Invitrogen Ltd). The thermal cycling protocol was 96°C for 2 min; 35 cycles of 96°C for 30 s, 51°C or 46°C for 30 s dependent on primer annealing characteristics, 72°C for 2 min; followed by 72°C for 10 min, in a MJ Research PTC-100 thermal cycler (Genetic Research Instrumentation Ltd). Successful PCR was confirmed by 1.5% (w/v) agarose gel electrophoresis [16]. PCR products for multiple individual microsatellite loci, amplified from aliquots of the same template DNA pool, were combined then purified using NucleoFast 96 PCR cleanup plates (Macherey-Nagel GmbH & Co. KG), before ligation into pDrive vector (Qiagen Ltd). Ligation products were diluted 1/10 with HPLC grade water and used as template for a second round of PCR using the 'reverse' microsatellite specific primer and a generic fluorescently labelled primer, M13 (-40), targeting the plasmid. Labelled amplicons were diluted 1/100 in HPLC grade water and fractionated by capillary electrophoresis on an ABI 3100 and viewed with genotyper 3.7 software (Applied Biosystems UK Ltd). The allele size reported by this method is that expected from the microsatellite primers plus an additional 150 bases of vector sequence. Comparison of profiles from pooled amplifications and those of constituent members of the pools demonstrated that homozygous individuals possessing a rare allele could be detected reliably in pools of 12 individuals or less. Thus, one strategy would be to assemble several small pools, allowing variance in allelic limits to be described, and continue screening until the addition of more pools no longer increases the allele range. In practice, however, it may be preferable to use much larger pools and accommodate for uncertainty over rare alleles by imposing buffer zones around detected allelic ranges prior to multiplexing. We empirically tested this approach. Template DNA from 96 diverse genotypes of *Theobroma cacao *was adjusted to 5 ng·uL^-1^, pooled and individually amplified by PCR for 84 dinucleotide cocoa microsatellite markers described by Pugh *et al *[15]. The complex profiles generated (Figure 1) broadly represent the array of alleles present when genotypes were assayed individually. We therefore selected 36 markers generating the widest range of homogeneous peaks for further study. The allelic range of peak sizes is taken to be indicative of the allelic range in the unsampled gene pool. In general, loci generating large numbers of peaks with approximately even height (Figure 1B,C) were highly informative whereas those producing few peaks (Figure 1A) or profiles dominated by one peak (Figure 1D) have less utility for genetic analysis.

We then examined the relationship between predicted allele range in pooled profiles and that observed after wider genotype sampling. For this, we employed multiplex PCR microsatellite analyses performed on 672 individual cocoa genotypes (Table 1). Two loci predicted to yield few alleles on the basis of the sample pool (mTcCIR080 and mTcCIR155) produced the same number of alleles when the sample range was expanded to include 672 individual cocoa genotypes. However, the number of alleles in more variable loci (mTcCIR131 and mTcCIR190) increased from 9 to 12 when the sample range was expanded, with the size range increasing by 67% and 62% respectively. Given a modest increase in allelic range when sample size was increased six fold, one approach would be to accommodate undetected alleles by imposing a buffer between the ranges of neighbouring loci prior to multiplexing. In this case, a spacing of 1× predicted range either side of the mean allele size would appear adequate. Overall, adoption of this protocol allows for improved selection of compatible polymorphic microsatellite markers, with reduced likelihood of producing overlapping profiles in multiplexed microsatellite reactions.

**Table 1:** Comparison of predicted and observed microsatellite allele frequency. The number of alleles each locus was predicted to generate as described in Figure 1 was compared to the actual alleles observed when screened over 672 genotypes of wild, uncultivated cocoa. Predictions were based on the height and number of peaks reported from the pooled samples and took account of the extra DNA amplified from the pDrive vector when predicting the size of the DNA fragments.

	PREDICTED				OBSERVED			
Locus	min	max	mode	alleles	min	max	mode	alleles
mTcCIR080	97	103	101	4	97	105	99	4
mTcCIR131	198	212	212	9	185	214	210	12
mTcCIR155	264	274	272	5	265	275	267	5
mTcCIR190	156	172	162	9	148	174	161	12
mTcCIR065	230	250	240	6	231	255	237	11
mTcCIR066	280	310	287	8	280	308	284	9
mTcCIR069	185	205	202	11	175	206	202	13
mTcCIR088	182	197	189	7	180	200	187	8
mTcCIR092	277	286	282	3	269	284	279	6
mTcCIR103	90	116	112	6	88	128	110	16
mTcCIR113	130	150	142	8	124	153	133	15
mTcCIR158	210	220	212	4	205	227	212	7
mTcCIR172	125	135	126	9	115	140	124	14
mTcCIR195	335	351	348	5	319	349	349	10
mTcCIR203	210	220	216	5	212	218	216	5
mTcCIR266	170	200	177	9	165	206	200	13

## Conclusion

Adoption of this methodology allows for both a qualitative and semi quantitative characterisation of polymorphism at individual microsatellite loci. When using pooled samples, combining DNA from up to 12 individuals allowed for the reliable detection of single copy alleles within that sample. If characterising microsatellite loci using DNA pools of greater than 12 individuals the incorporation of a buffer zone into the final genotyping assay, based on the observed range of allele sizes, can allow for the variability likely to be encountered in a larger sample size. The methodology is suitable for high throughput applications by the combination of differing fluorescent dyes in association with convenient liquid handling formats. This protocol benefits from initially utilising unlabelled primers identical to those used in the final genotyping assay, reducing the possibilities of unexpected banding patterns due to changes in primer sequence or assay conditions. The high resolution DNA size measurement makes this protocol suitable for characterising dinucleotide microsatellite loci.

## List of abbreviations

PCR, Polymerase chain reaction; DNA, Deoxyribonucleic acid; ng, 10^-9 ^gram

## Competing interests

The author(s) declare that they have no competing interests.

## Authors' contributions

NCC conceived and developed the microsatellite selection protocol and drafted the manuscript. The overall project was conceived by DRB. MJW aided the experimental design and played a major role in developing the manuscript. All authors have read and approved the final manuscript.
